# The Pupillary Light-Off Reflex in Acute Disorders of Consciousness

**DOI:** 10.1007/s12028-024-02133-9

**Published:** 2024-09-25

**Authors:** Pardis Zarifkar, Marwan H. Othman, Karen Irgens Tanderup Hansen, Moshgan Amiri, Sarah Gharabaghi Stückler, Maria Louise Fabritius, Sigurdur Thor Sigurdsson, Christian Hassager, Peter F. Birkeland, John Hauerberg, Kirsten Møller, Jesper Kjaergaard, Merlin D. Larson, Daniel Kondziella

**Affiliations:** 1 Department of Neurology, Rigshospitalet, Copenhagen University Hospital, Inge Lehmanns Vej 8, 2100 Copenhagen, Denmark; 2 Department of Neuroanaesthesiology, Rigshospitalet, Copenhagen University Hospital, Copenhagen, Denmark; 3 Department of Cardiology, Rigshospitalet, Copenhagen University Hospital, Copenhagen, Denmark; 4 Department of Clinical Medicine, University of Copenhagen, Copenhagen, Denmark; 5 Department of Neurosurgery, Rigshospitalet, Copenhagen University Hospital, Copenhagen, Denmark; 6 Department of Anesthesiology, University of California San Francisco, San Francisco, CA USA

**Keywords:** Brain injury, Coma, Consciousness, Neuromonitoring, Pupillometry, Prognostication

## Abstract

**Background:**

In intensive care patients with disorders of consciousness, the pupillary light reflex is a measure of pupillary parasympathetic function. By contrast, the pupillary light-off reflex leads to pupil dilation in response to an abrupt change from light to darkness (“light-off”) and reflects combined parasympathetic and sympathetic pupillary function. To our knowledge, this reflex has not been systematically investigated in patients with disorders of consciousness. We hypothesized that the pupillary light-off reflex correlates with consciousness levels after acute brain injury.

**Methods:**

From November 2022 to March 2023, we enrolled 100 study participants: 25 clinically unresponsive (coma or unresponsive wakefulness syndrome) and 25 clinically low-responsive (minimally conscious state or better) patients from the intensive care units of a tertiary referral center, and 50 age-matched and sex-matched healthy controls. Exclusion criteria were active or chronic eye disease. We used automated pupillometry to assess the pupillary light-off reflex and the pupillary light reflex of both eyes under scotopic conditions in all study participants.

**Results:**

The pupillary light-off reflex was strongly correlated with consciousness levels (*r* = 0.62, *p* < 0.001), the increase in pupillary diameters being smallest in unresponsive patients (mean ± standard deviation 20% ± 21%), followed by low-responsive patients (mean ± standard deviation 47% ± 26%) and healthy controls (mean ± standard deviation 67% ± 17%; *p* < 0.001). Similar yet less pronounced patterns were observed for the pupillary light reflex. Twenty-one of 25 (84%) unresponsive patients had preserved pupillary light reflexes, but only seven (28%) had fully preserved pupillary light-off reflexes (*p* < 0.0001). Of these 7 patients, five (71%) regained awareness.

**Conclusions:**

The pupillary light-off reflex may be more sensitive to consciousness levels than the pupillary light reflex. The clinical implications of this finding seem worthy of further investigation, particularly regarding possible benefits for neuromonitoring and prognostication after brain injury.

**Supplementary Information:**

The online version contains supplementary material available at 10.1007/s12028-024-02133-9.

## Introduction

The pupillary light reflex is a continuous physiological response that optimizes the amount of light reaching the retina, indicating integrity of the parasympathetic pupillary pathways [1], and it is an essential component of neuromonitoring and prognostication in intensive care [2]. Unlike the pupillary light reflex, the pupils’ reactions to darkness are much less well known because these responses are impossible to see with the unaided eye and require infrared-sensitive motion recordings.

**Fig. 1:**
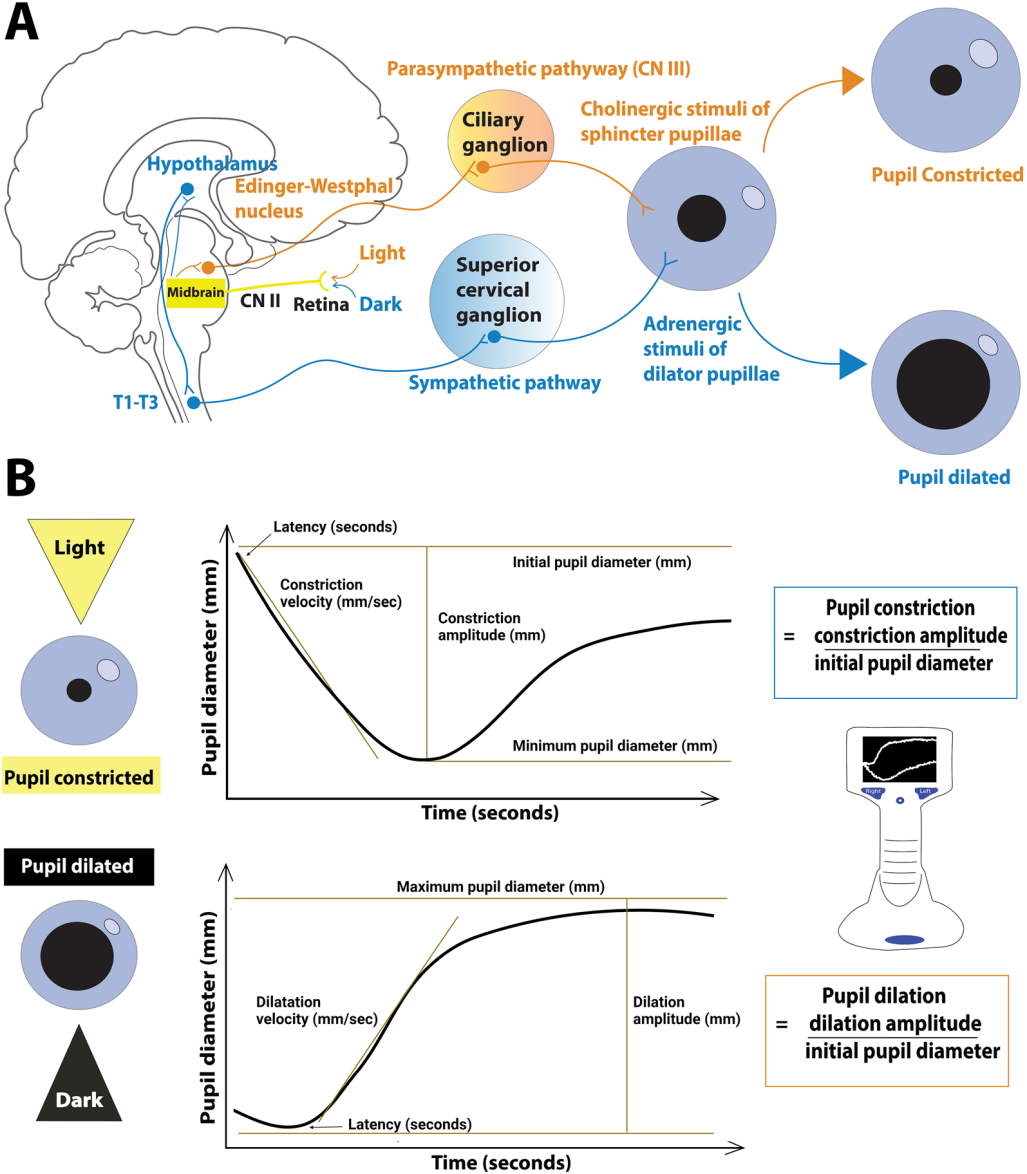
Simplified illustration of some of the parasympathetic and sympathetic pathways that regulate **a** pupil size and **b** measurement of the pupillary light and light-off reflexes using automated pupillometry. When exposed to light, retinal cells send signals through the optic nerve to the pretectal nucleus, which communicates with the Edinger–Westphal nucleus. Parasympathetic fibers travel via the oculomotor nerve to the ciliary ganglion and the sphincter pupillae muscle, causing pupil constriction (pupillary light reflex). In darkness, the retina signals the hypothalamus, activating the sympathetic pathway via the spinal cord (T1-T2), leading to the superior cervical ganglion. Postganglionic fibers then travel to the dilator pupillae muscle, resulting in pupil dilation (pupillary light-off reflex). The pupillary light reflex is the difference between initial pupillary diameter and the minimal pupillary diameter after constriction, divided by the initial pupil diameter. By contrast, the pupillary light-off reflex is the difference between the initial pupillary diameter and the maximal pupillary diameter after dilatation, divided by the initial pupil diameter. A change in pupillary diameter during pupillary dilation or constriction is defined as the increase or decrease in pupillary diameter (curve amplitude) relative to the initial diameter. It should be noted that the figure omits the contribution of the inhibitory effect on the Edinger–Westphal nucleus and that the anatomical pathways mediating the pupillary light-off reflex are not yet entirely understood

In her seminal work on the pupil, Irene Loewenfeld [3] distinguished the pupillary light-off reflex and the pupillary darkness reflex. Both reflexes involve pupil dilation in response to darkness, but the pupillary darkness reflex is merely a short-lived response to a brief dark pause (“dark flash”) with the light reappearing immediately thereafter. By contrast, “after full retinal bleaching by a powerful light, the pupillary light-off reflex begins forcefully and smoothly; but after a few seconds in darkness, the movement slows down [and] begins to waver….” Therefore, it should be noted that the pupillary light-off reflex is also different from the simple relaxation part of the pupillary light reflex because it occurs only with an intense and prolonged light stimulus preceding the “light-off.” Both sympathetic excitation and parasympathetic relaxation participate in the pupillary light-off response [3]. Hence, the pupillary light-off reflex is brought about through inhibition of the Edinger–Westphal nucleus and activation of the sympathetic dilator muscle [4–6]. Psychosensory stimuli, such as sudden noise or squeezing the back of the neck, may enhance the pupillary light-off reflex [6]. Disruptions to the pupillary light-off reflex can occur with lesions that affect the sympathetic nervous system, even when the pupillary light reflex remains intact [3, 4, 6, 7]. Figure 1 provides a simplified overview of the pupillary light and the pupillary light-off reflexes.

Because acute brain injury often involves diffuse damage to multiple neural circuits, the pupillary light-off reflex may provide additional information about autonomic and higher-order brain functions after brain injury compared with the pupillary light reflex [8–10]. However, the pupillary light-off reflex is not clinically routine in the intensive care unit (ICU) and to our knowledge, it has not previously been studied in patients with disorders of consciousness (DoC) with acute brain injury.

We investigated the pupillary light-off reflex of patients with DoC who were clinically unresponsive (coma or unresponsive wakefulness syndrome [UWS]) or clinically low-responsive (minimally conscious states [MCS]) and healthy controls. We hypothesized that the presence and strength of the pupillary light-off reflex is correlated with levels of consciousness impairment after brain injury and that these correlations are similar to those of the pupillary light reflex, but not necessarily identical.

## Methods

### Participants

We prospectively enrolled patients on a convenience base from two ICUs (neurological/neurosurgical and cardiologic) at Rigshospitalet, Copenhagen University Hospital, a tertiary referral center. Inclusion criteria were age ≥ 18 years, acute traumatic or nontraumatic brain injury, and impaired consciousness, as previously described [11–14]. Age-matched (± 5 years) and sex-matched healthy controls were recruited from the community through a word-of-mouth advertisement. Written informed consent was obtained from all participants or their surrogate decision-makers.

### Clinical Examination

Patients were examined during the daytime between 10:00 and 15:00 and when deemed feasible by the attending medical staff. Neurological assessments of consciousness were performed as described earlier [11–14], including three clinical scales: the Glasgow Coma Scale, the Full Outline of UnResponsiveness score [15], and the Simplified Evaluation of Consciousness Disorders [16, 17]. Patients with a coma or UWS were categorized as clinically unresponsive, and patients in MCS or emerged from MCS were classified as being clinically low-responsive [18]. Individuals with active eye diseases or a history of eye injury were excluded. Clinical data, such as medical history, admission etiology, diagnosis, and sedation, were extracted from electronic health records. Sedation levels were stratified as described earlier into none, low to moderate (fentanyl < 500 µg/h, remifentanil < 1000 µg/h, propofol < 100 mg/h, midazolam < 10 mg/h, sevoflurane < 3%, or equivalent combinations), and high to very high (fentanyl ≥ 500 µg/h, remifentanil ≥ 1000 µg/h, propofol ≥ 100 mg/h, midazolam ≥ 10 mg/h, sevoflurane ≥ 3%, any dosage of sodium thiopental, or equivalent combinations) [12–14].

### Pupillometry

Pupillometry was conducted once for every patient. Pupillary reflexes were investigated under scotopic, or low-light, conditions, as described in our previous publications [11, 19]. Before pupillometry assessments, we reduced ambient light levels to below 10 lux by closing curtains and turning off ambient lights, which was monitored using the LUX Light Meter app on a conventional iPhone. We used the NeurOptics PLR-3000 pupillometer to measure the pupillary light-off reflex by creating a bright light, which was then turned off. The light source was directed at the pupil for at least 3 s before initiating the measurement sequence. After the initial pulse, the light was deactivated, and the pupil was continuously measured for an additional 7 s. The protocol entailed a negative pulse stimulus (pulse intensity = 0 µW, background intensity = 180 µW, total measurement duration = 8.02 s, including pulse duration = 7.02 s, and pulse onset = 1.00 s). We subsequently used the NeurOptics NPi-200 Pupillometer to assess the pupillary light reflex in both eyes. For both pupillary light and pupillary light-off reflexes, we investigated changes in pupillary diameter, dilation velocity, and latency among unresponsive patients, low-responsive patients, and healthy controls. A change in pupillary diameter during pupillary dilation or constriction was defined as the increase or decrease in pupillary diameter (curve amplitude) relative to the initial diameter: (maximum pupillary diameter−initial pupillary diameter)/initial pupillary diameter (Fig. 1). In addition, we quantified the pupillary light reflex using the Neurological Pupil index (NPi), which ranges from 0 (nonreactive) to 5 (normal), with an NPi score of 3 or higher being considered within normal physiological limits [1]. All data underwent visual inspection to identify and remove anomalies, for example, blinking artifacts, as described earlier [13].

### Outcomes

The primary outcomes were the magnitude and reactivity of the pupillary light-off reflex assessed in both eyes of unresponsive and low-responsive patients with DoC and a comparison between patients with DoC and healthy controls. Secondary outcomes included (1) the correlation between the pupillary light-off reflex and levels of consciousness impairment in patients, (2) differences in the pupillary light reflex among all groups, and (3) the proportion of patients exhibiting discordance between pupillary light-off and light reflexes.

### Statistical Analyses

Differences in demographic and pupillometry characteristics among unresponsive and low-responsive patients and healthy controls were assessed using a *χ*^2^ test or Fisher’s exact test for categorical data and a one-way analysis of variance with a post hoc Tukey test to account for multiple comparisons for numeric data. Comparisons between unresponsive and low-response patients were made using Student’s *t*-test, Mann–Whitney *U*-test, or Fisher’s exact test, as appropriate. A significant pupillary light reflex was defined as an NPi score of 3 or higher [1]. In the absence of a specific cut-off for a normal or fully preserved pupillary dilation during the light-off reflex, this was defined as being within two standard deviations of the mean pupillary dilation response (percentage change from initial size) observed in healthy controls. McNemar’s test was used to analyze the discordance between binary pupillary light-off and pupillary light reflexes in unresponsive patients. Spaghetti, scatter, and box plots were used to visualize initial and final pupillary diameters during pupillary reflex measurements. The association between mean pupillary dilation (relative change across left and right eyes) and consciousness levels (coma, UWS, MCS, and fully conscious) across all study participants was assessed using Spearman’s Rank correlation coefficient and an ordinal regression model. Missing data were reported and excluded from the analyses. All analyses were conducted using R statistical software v. 4.3.2 (R Core Team, Vienna, Austria).

## Results

Between November 2022 and March 2023, we enrolled 100 study participants, including 50 patients with DoC and 50 healthy controls. Among the patients with DoC, 25 were clinically unresponsive, and 25 were low-responsive. Demographic and clinical characteristics are provided in Table 1. There were no significant differences between the patient groups in terms of age (F [2, 97] = 0.31, *p* = 0.73) or sex (*χ*^2^ = 1.39, *p* = 0.50). In both patient groups, hemorrhagic stroke was the leading cause of brain injury (52% in unresponsive and 32% in low-responsive patients), followed by traumatic brain injury (20%) in unresponsive patients and cardiovascular causes (28%) in low-responsive patients.

**Table 1:** Demographic and clinical characteristics

Characteristic	Unresponsive patients*	Low-responsive patients*	Healthy controls
Number of participants	25	25	50
Age (mean ± SD)	59.8 ± 16.6	63.2 ± 13.0	62 ± 15
Male sex, n (%)	18 (72%)	14 (56%)	32 (64%)
Premorbid mRS 0–2, n (%)	19 (76%)	24 (96%)	–
Days since injury (mean ± SD)	7 ± 6	8 ± 9	–
GCS (median, IQR)	3 (3, 5)	14 (11, 15)	–
FOUR (median, IQR)	4 (3, 5)	16 (13, 16)	–
Consciousness categories, n (%)	Coma: 22 (88%) UWS: 3 (12%)	MCS: 10 (40%) eMCS: 15 (60%)	–
Brain injury etiology, n (%)			
Subarachnoid hemorrhage	3 (12%)	5 (20%)	–
Intracerebral hemorrhage	10 (40%)	3 (12%)	–
Ischemic stroke	2 (8%)	1 (4%)	–
Traumatic brain injury	5 (20%)	1 (4%)	–
Ischemic-anoxic brain damage of cardiac origin	3 (12)	7 (28)	–
Other	2 (8%)^#^	8 (32%)^##^	–
Airway management, n (%)			
Noninvasive	2 (8%)	16 (64%)	–
Tracheostomy	4 (16%)	3 (12%)	–
Oral intubation	19 (76%)	6 (24%)	–
Sedation**			
None to minimal	9 (36%)	21 (84%)	–
Low to moderate	1 (4%)	2 (8%)	–
High to very high	15 (60%)	2 (8%)	–

^*^Patients categorized as unresponsive were in a coma or unresponsive wakefulness syndrome; those categorized as low-responsive were in the minimally conscious state or better (including patients emerged from the minimally conscious state)

^**^See methods for details about sedation

^#^Status epilepticus, herpes simplex encephalitis

^##^Carbon monoxide poisoning, nonconvulsive status epilepticus following cerebral shunt malfunction, respiratory arrest due to Guillain-Barré, respiratory arrest due to myasthenia gravis, cerebral tumor and three aorta dissections

eMCS = emerged from MCS, IQR–interquartile range, MCS = minimally conscious state, SD = standard deviation, UWS = unresponsive wakefulness syndrome

### Pupillary Light-Off and Pupillary Light Reflexes Indices

**Fig. 2:**
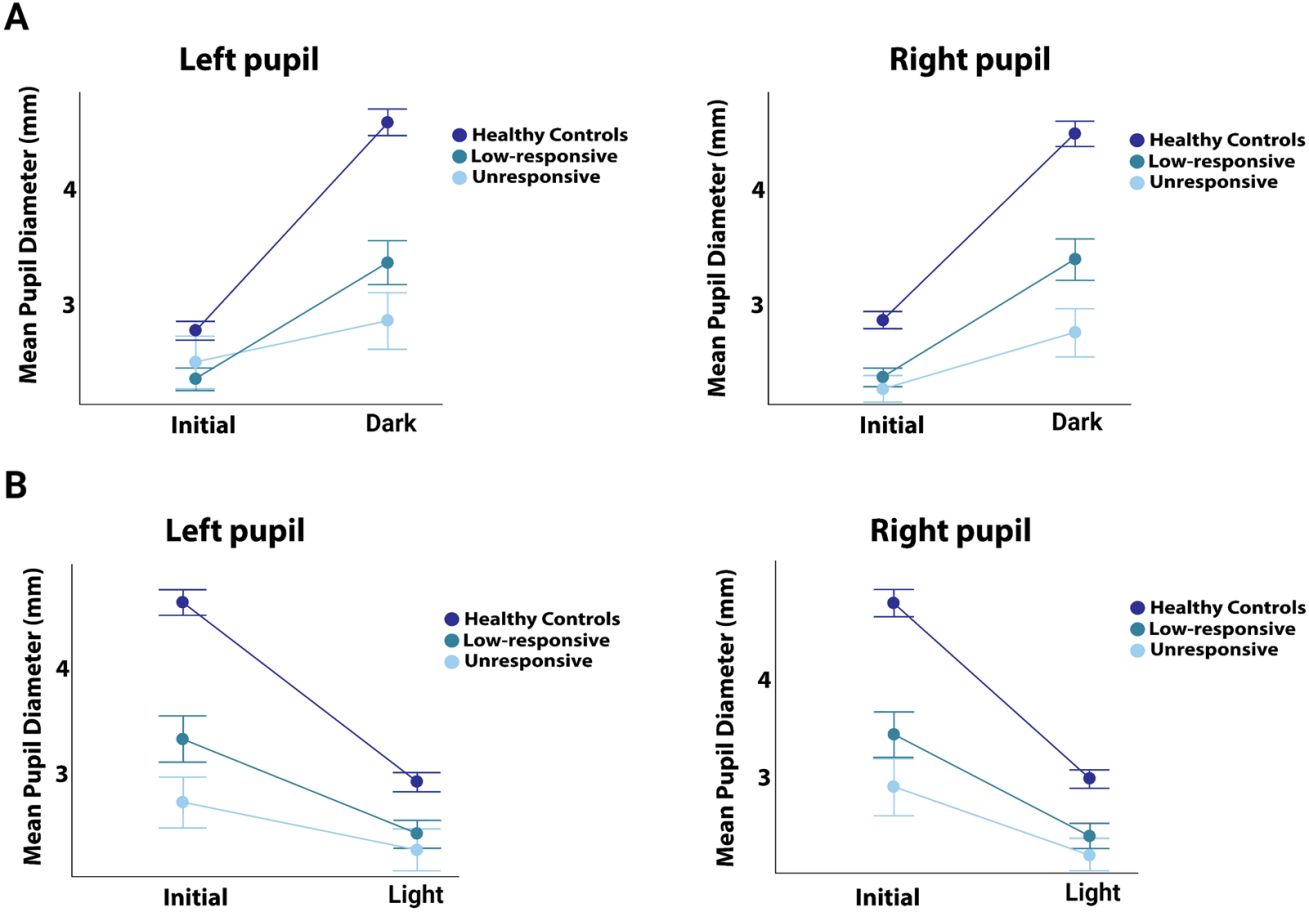
Pupillary diameter changes as a function of **a** the pupillary light-off reflex and **b** the pupillary light reflex. The figures illustrate changes in mean pupillary diameter during the pupillary light-off and light reflexes for unresponsive patients, low-responsive patients, and healthy controls in left and right eyes. Patients exhibited significantly smaller increases and decreases in pupillary diameter compared to healthy controls, with unresponsive patients showing the smallest changes*.* Like the pupillary light reflex, the pupillary light-off reflects distinguishes well between clinically unresponsive and low-responsive patients. For individual pupillary diameter data see Fig. 3

**Fig. 3:**
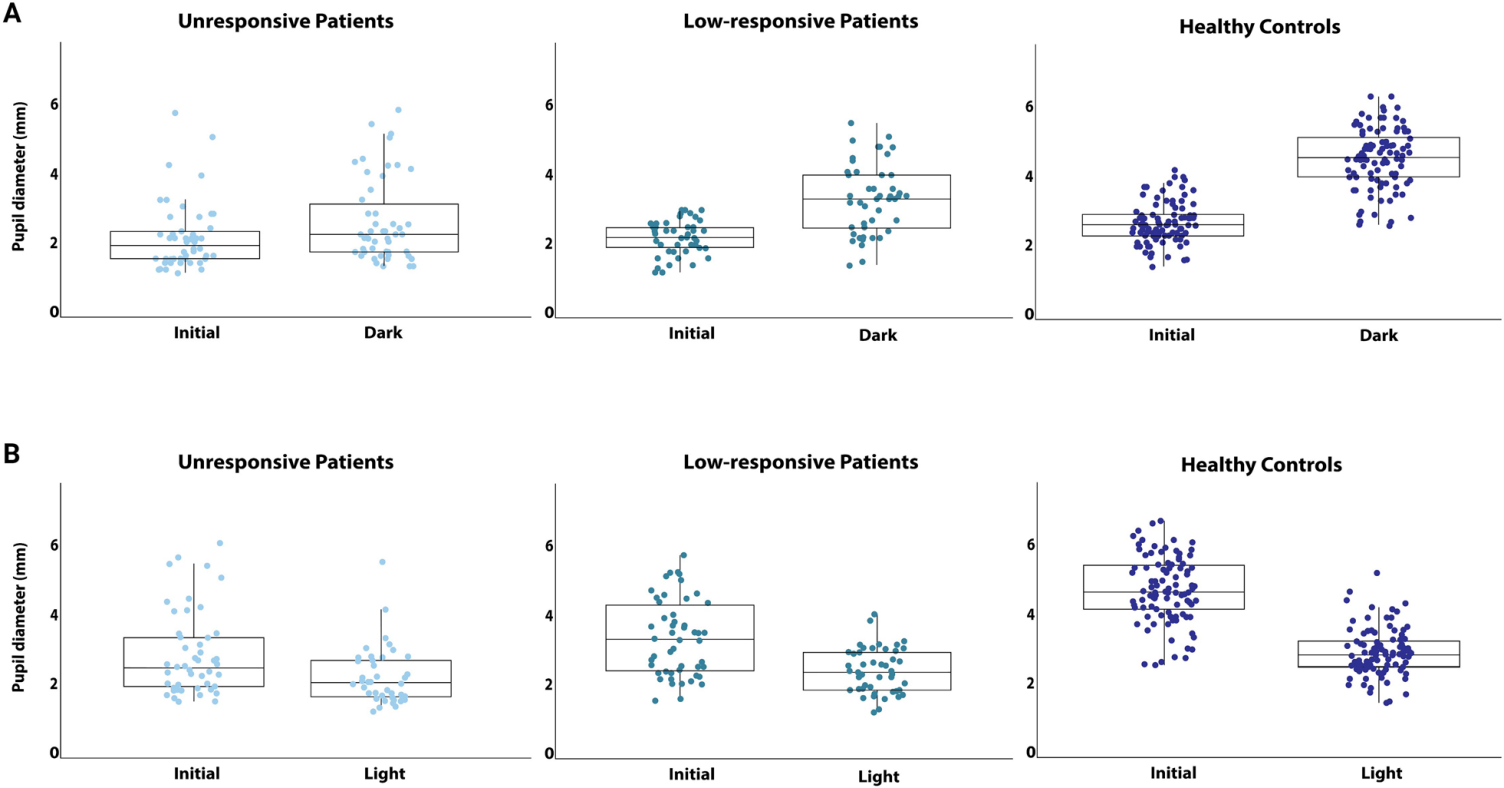
Individual pupillary diameter changes during **a** the pupillary light-off reflex and **b** the pupillary light reflex for clinically unresponsive patients, low-responsive patients, and healthy controls, displayed as scatter plots with overlaying box plots

Data regarding the pupillary light-off reflex are summarized in Table 2. During the pupillary light-off reflex, the absolute and relative increase in pupillary diameter was smallest in unresponsive patients (mean ± SD: 0.5 mm ± 0.6 mm or 20% ± 21%), followed by low-responsive patients (1.1 mm ± 0.7 mm or 47% ± 26%) and healthy controls (1.8 mm ± 0.5 mm or 67% ± 17.4%). This difference in pupillary dilation was significant across the groups (F [2, 97] = 43.1, *p* < 0.001). Specifically, the increase in pupillary diameter was smaller in unresponsive patients compared to low-responsive patients (mean difference between groups, M = −20, *p* < 0.001) and healthy controls (M = −47, *p* < 0.001) and smaller in low-responsive patients compared with healthy controls (M = −27, *p* < 0.001). Figures 2a and 3a provide details. Significant differences in dilation velocity were observed for the left (but not the right) pupil across groups (F [2, 86] = 6, *p* = 0.004), with unresponsive patients showing slower dilation velocity compared to healthy controls (M = −1.1, *p* = 0.006). There were no differences in latency to dilation across any groups.

**Table 2:** Pupil diameter changes during the pupillary light-off reflex

	Unresponsive patients*	Low-responsive patients*	Healthy controls	*P* value**
Initial diameter (mm)	L: 2.5±1.2 R: 2.2±0.7	L: 2.3±0.5 R: 2.3±0.4	L: 2.8±0.6 R: 2.8±0.6	L: 0.05 R: **<0.001**
End diameter (mm)	L: 2.8 ± 1.3 R: 2.7 ± 1.2	L: 3.4 ± 1.0 R: 3.4 ± 1.0	L: 4.6 ± 0.9 R: 4.6 ± 0.9	L: **< 0.001** R: **< 0.001**
Change in pupillary diameter: dilation (mm)	L: 0.4 ± 0.5 R: 0.5 ± 0.7	L: 1.0 ± 0.7 R: 1.1 ± 0.7	L: 1.9 ± 0.5 R: 1.8 ± 0.5	L: **< 0.001** R: **< 0.001**
Relative change in pupillary diameter: dilation (%)	L: 16.6 ± 21.9 R: 23 ± 24.7	L: 44.5 ± 28.5 R: 49.5 ± 28.8	L: 68.9 ± 18.8 R: 65 ± 20.0	L: **< 0.001** R: **< 0.001**
Dilation velocity (mm/sec)	L: 0.3 (0.1–1.2) R: 0.4 ± 0.2	L: 0.6 (0.3–1.6) R: 1.2 ± 1.2	L: 1.0 (0.42–10.2) R: 1.5 ± 2.1	L: **0.004** R: 0.07
Latency (sec)	L: 0.39 ± 0.13 R: 0.39 ± 0.19	L: 0.37 ± 0.12 R: 0.46 ± 0.20	L: 0.45 ± 0.13 R: 0.40 ± 0.12	L: **0.048** R: 0.24

Pupillometry results are presented as mean ± standard deviation

^*^Patients categorized as unresponsive were in a coma or unresponsive wakefulness syndrome; those categorized as low-responsive were in the minimally conscious state or better

^**^One-way ANOVA test results. Bolded values indicate significant differences

L, left; R, Right

Similar patterns were observed for the pupillary light reflex (Table 3, Figs. 2b and 3b). The decrease in pupillary diameter was smaller in unresponsive patients (M = 17.5 ± 10.6%) compared to low-responsive patients (M = 27 ± 10.5%) and healthy controls (M = 37.5 ± 5.5%). This difference in pupillary constriction was significant across all groups (F [2, 93] = 46, *p* < 0.001). Significant differences in NPi scores were also observed (F [2, 97] = 12.3, *p* < 0.001). NPi scores were lower in unresponsive patients (3.3 ± 1.7) than in low-responsive patients (4.3 ± 0.6, *p* < 0.001) and healthy controls (4.4 ± 0.4, *p* < 0.001), but there was no significant difference between low-responsive patients and healthy controls. Constriction velocity varied significantly across groups (F [2, 93] = 45, *p* < 0.001), being smallest in unresponsive patients (1.0 ± 0.7), followed by low-responsive patients (1.7 ± 0.9) and healthy controls (2.6 ± 0.6). There were no significant differences in latency to constriction (F [2, 92] = 0.09, *p* = 0.092).

**Table 3:** Pupil diameter changes during the pupillary light reflex

	Unresponsive patients*	Low-responsive patients*	Healthy controls	*P* value**
Initial diameter (mm)	L: 2.9 ± 1.2 R: 2.8 ± 1.2	L: 3.3 ± 1.1 R: 3.4 ± 1.2	L: 4.7 ± 0.9 R: 4.8 ± 1.0	L: **< 0.001** R: **< 0.001**
End diameter (mm)	L: 2.3 ± 0.9 R: 2.2 ± 0.7	L: 2.4 ± 0.7 R: 2.4 ± 0.6	L: 2.9 ± 0.6 R: 3.0 ± 0.7	L: **< 0.001** R: < **0.001**
Change in pupillary diameter: constriction (mm)	L: 1.0 ± 1.4 R: 1.1 ± 1.1	L: 0.9 ± 0.6 R: 1.0 ± 0.7	L: 1.7 ± 0.4 R: 1.8 ± 0.5	L: **<0.001** R: **<0.001**
Relative change in pupillary diameter: constriction (%)	L: 16 ± 12 R: 21 ± 12	L: 26 ± 10 R: 28 ± 12	L: 37 ± 5.6 R: 38 ± 6.0	L: **< 0.001** R: < **0.001**
Constriction velocity (mm/sec)	L: 0.9 ± 0.8 R: 1.2 ± 0.8	L: 1.7 ± 0.9 R: 1.7 ± 0.9	L: 2.6 ± 0.6 R: 2.6 ± 0.6	L: < **0.001** R: < **0.001**
Latency (sec)	L: 0.25 ± 0.12 R: 0.25 ± 0.04	L: 0.25 ± 0.05 R: 0.24 ± 0.05	L: 0.24 ± 0.03 R: 0.24 ± 0.04	L: 0.72 R: 0.92
NPi	L: 3.3 ± 1.7 R: 3.2 ± 1.9	L: 4.3 ± 0.7 R: 4.4 ± 0.6	L: 4.4 ± 0.4 R: 4.4 ± 0.4	L: **< 0.001** R: < **0.001**

Pupillometry results are presented as mean ± standard deviations

^*^Patients categorized as unresponsive were in a coma or unresponsive wakefulness syndrome; those categorized as low-responsive were in the minimally conscious state or better

^**^One-way ANOVA test results

Bolded values indicate significant differences

L, left; NPi, Neurological Pupil index; R, Right

### Pupillary Light-Off and Pupillary Light Reflexes in DoC in the ICU

The association between pupillary reflexes and consciousness levels was assessed using Spearman's rank correlation coefficient and an ordinal regression model. Spearman's rank correlation was 0.62 (*p* < 0.001) for the pupillary light-off reflex, indicating a positive association between greater pupillary dilation and higher consciousness levels. The ordinal regression model confirmed this, showing a significant association between greater pupillary dilation and higher consciousness levels (estimate = 0.051, SE = 0.013, *p*<0.001). This association remained significant after accounting for sedation, with higher consciousness levels linked to greater pupillary dilation (estimate = 0.032, SE = 0.01, *p* = 0.021) and increased sedation linked to lower consciousness levels (estimate = −1.24, SE = 0.45, *p* = 0.006). There was a similar, but less pronounced association between the pupillary light reflex and consciousness levels (r = 0.39, *p* = 0.005; estimate = 0.089, SE = 0.35, *p* = 0.02), which remained significant when accounting for sedation (estimate = 1.02, SE = 0.37, *p* = 0.006).

**Fig. 4:**
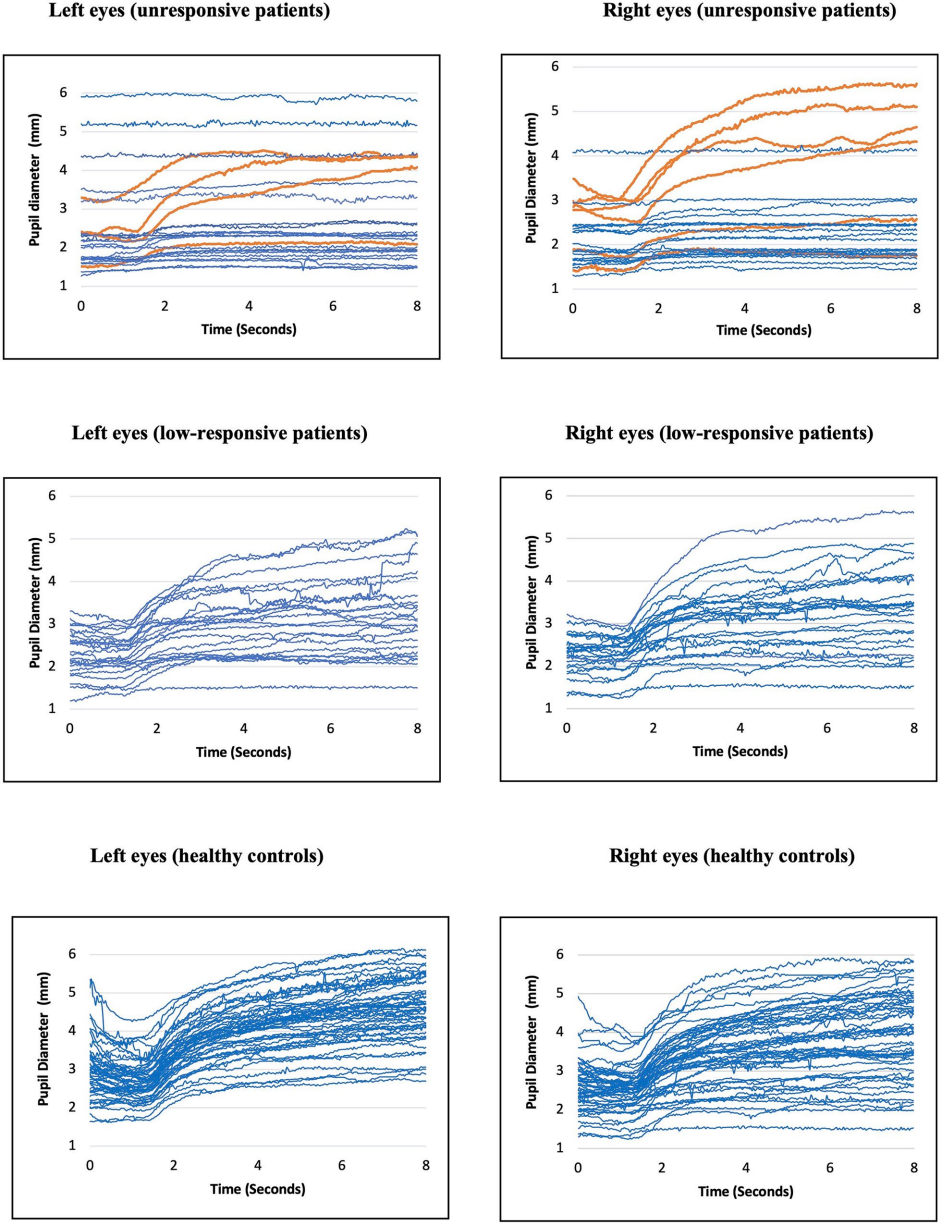
Raw data of the pupillary light-off reflex of clinically unresponsive and low-responsive patients, and age-and sex-matched healthy controls, illustrating pupillary diameters over time. Time in seconds is displayed on x-axes, and pupillary diameters in millimeters are shown on y-axes. Preserved light-off reflexes of unresponsive patients, as defined by pupillary dilation within two standard deviations of the mean pupillary dilation response observed in healthy controls, are highlighted in orange. Note that rarely, a rudimentary pupillary dilation response appears in unresponsive patients that is outside the normal range. The figure shows that the shape and distribution of the pupillary light-off reflex correlate with increasing consciousness levels, i.e., from mostly absent in clinically unresponsive patients to brisk in healthy volunteers

Among unresponsive patients, three of 25 (12%) exhibited bilaterally preserved pupillary light-off reflexes, while an additional four patients (16%) had a pupillary light-off reflex in one eye (Fig. 4). The clinical and pupillometry characteristics of the 7 patients with a unilaterally or bilaterally preserved pupillary light-off reflexes are detailed in Table S1. All these 7 patients were male; four had hemorrhagic stroke, two had traumatic brain injury, and one had post-cardiac arrest ischemic-anoxic brain damage. Four patients received high to very high levels of sedation, while three received none to minimal. Except for 2 patients diagnosed with UWS, all were in a coma and orally intubated or tracheostomized at the time of measurement. All seven unresponsive patients with preserved pupillary light-off reflexes also had preserved pupillary light reflexes in the eye(s) with a pupillary light-off reflex. More unresponsive patients had preserved pupillary light reflexes than pupillary light-off reflexes: 18 of 25 (72%) had bilateral light reflexes, while an additional three (12%) had pupillary light reflexes in one eye. McNemar's test indicated a significant discordance of 60% between pupillary light-off and light reflexes in the unresponsive patient group (*p* < 0.001). In sum, 21 of 25 (84%) clinically unresponsive patients had preserved pupillary light reflexes, but only seven (28%) had preserved pupillary light-off reflexes (Fisher exact test, *p* value < 0.0001).

In the low-responsive patient group, pupillary reflexes were more commonly preserved. Nineteen of 25 (76%) low-responsive patients had preserved pupillary light-off reflexes (16 bilaterally, two only in the left eye, and one only in the right eye). All 25 (100%) low-responsive patients had preserved pupillary light reflexes, resulting in a nonsignificant discordance of 28% between the light and light-off reflexes (*p* = 0.131). However, while the pupillary light-off reflex was able to show a difference between the low-responsive patients and healthy controls (Table 2), the NPi was not (Table 3).

Three months after assessment, 23 of 25 unresponsive patients and 24 of 25 low-responsive patients could be followed up. Fourteen of 23 (75%) unresponsive patients and 21 of 24 (88%) low-responsive patients survived (Fisher’s exact test, *p* = 0.049). Six of the seven unresponsive patients with a preserved pupillary light-off reflex in at least one pupil were available for follow-up: Five regained awareness, as measured by patients’ ability to respond to internal and environmental stimuli, with the first signs of clinical awareness appearing after 9 ± 5 days. In 4 of these 5 patients, the first sign of awareness was visual tracking as noted by the attending clinicians. The one patient who did not regain consciousness had life-supporting treatment withdrawn on the same day as the pupillometry.

## Discussion

Our study demonstrates that the pupillary light-off reflex is diminished in patients with DoC with acute brain injury, especially in those who are clinically unresponsive, and that this reflex correlates with the levels of consciousness. We have shown that the pupillary light-off reflex may be abolished or abnormal in patients with preserved pupillary light reflexes, thereby potentially adding prognostic information in patients with acute DoC in the ICU.

### The Pupillary Light-Off Reflex Compared to the Pupillary Light Reflex in Patients with DoC

Both relaxed and dilated pupillary diameters were smaller in patients with DoC compared to healthy controls, with the lowest increase in pupillary dilation and number of preserved light-off reflexes observed in unresponsive patients, followed by low-responsive patients. Although the pupillary light reflex showed similar patterns, these appeared to be less pronounced than what we found for the pupillary light-off reflex, suggesting that sympathetic input to the pupils is overall more severely compromised than parasympathetic input. While the exact reasons require further investigations, these findings are consistent with previous research: We recently showed that eye drops containing brimonidine, an alpha-2 agonist reducing sympathetic pupillary tone, do not significantly change pupil size in deeply comatose patients, indicating the absence of sympathetic innervation [11]. The present study extends these findings by demonstrating the autonomic nervous system’s influence (or lack thereof) on the association of the pupillary light-off reflex with levels of consciousness.

Why might the pupillary light-off reflex provide more information than just measuring pupil size in the dark? When light is turned off, the pupil dilates quickly, but it does not reach maximum size until about 50–60 s after darkness [3]. So, when the lights in the patient room are turned out and we go to study the patient, the time is variable, and the size of the pupil when recording the NPi depends on the amount of time that elapses. The light-off reflex is more precise because the measurement starts with a known, stronger, and longer lasting light intensity and each measurement is carefully timed (7 s of “light-off”). This provides a more precise measurement that can be trended over time and thus may follow the degree of consciousness more precisely. Indeed, while the NPi was unable to show a difference between the low-responsive patients and healthy controls, the pupillary light-off reflex did show this difference. Furthermore, among the 25 clinically unresponsive patients, only three had preserved pupillary light-off reflexes bilaterally and four unilaterally, all of whom had preserved light reflexes. Automated pupillometry assessing the pupillary light-off reflex also appears to distinguish between clinically unresponsive and low-responsive patients with better precision than some other technological approaches that require much greater computational expertise like, for example, arterial spin labeling MRI evaluating cerebral blood flow measurements [20].

Of the six unresponsive patients with a pupillary light-off reflex who could be followed up, five regained clinical awareness. Interestingly, it has been argued that sympathetic pupillary innervation requires consciousness to be preserved to at least some degree [11], and the proportion of clinically unresponsive patients with a preserved pupillary light-off reflex is of the same order of magnitude as the proportion of unresponsive patients with covert consciousness (15–25%) [13, 21–24]. In future studies it will be interesting to investigate whether the pupillary light-off reflex could be a sign of covert consciousness and help to identify clinical trajectories after brain injury [25].

### Uncertainties About the Pupillary Light-Off Reflex in Terms of Physiology and Pathophysiology

The exact physiologically underpinnings of the pupillary light-off reflex are not fully understood. It must be noted, as stated earlier, that the light-off dilation is not entirely sympathetically generated. As Omary has demonstrated [26], the pupillary light-off reflex may still be present in patients with Horner’s syndrome. Loewenfeld discusses how “light-off” activates neurons in the pretectal nucleus that are then inhibitory to the Edinger–Westphal nucleus and argues that the pupillary light-off reflex is a combination of sympathetic activation and inhibition of the parasympathetic nucleus [3]. Sillito et al. shows how the pupillo-constrictor neurons are inhibited at “light off” [27]. Loewenfeld also discusses a pathway from the retina to the suprachiasmatic nucleus [3]. That nucleus increases the activity of the preganglionic sympathetics at “light-off.” Pupillary dilation then follows as the sympathetic tone is augmented. Although this pathway might not be present in primates [3], it is important to realize that the anatomical pathways of the pupillary light-off reflex outlined in Fig. 1 probably are not complete, but a proper diagram is not yet available. Samuels and Szabadi also show a complex figure, but the pathways they outline are not proven [28].

Furthermore, in terms of pathophysiology, future research must meticulously investigate the effects of opioids and general anesthesia on the pupillary light-off reflex and how this reflex may reemerge (or not) when sedation is stopped in people with acute brain injury. Opioids appear effective in blocking pupillary reflex dilation (which is not the same as the pupillary light-off reflex) without altering the light reflex [29]. As opioids block inhibitory pathways into the Edinger–Westphal nucleus [30], do unresponsive patients lack a pupillary light-off reflex because of the drugs or because of central nervous system injury? Of the seven unresponsive patients with a preserved pupillary light-off reflex, four received high to very high levels of sedation, showing that at least in a subset of sedated patients the pupillary light-off reflex remains intact. Complicating this matter further, however, tolerance to the effects of opioids on the pupil does occur [31]. The effects of opioids on the pupillary light-off reflex after brain injury may thus change over time and perhaps with the etiology of the brain injury. Although we accounted for sedation in the statistical analysis, all these considerations must be addressed by replication studies with serial pupillary measurements of DoC cohorts with large enough numbers to account for the heterogeneity of sedation and brain injury etiologies.

### Strength and Limitations

This study has several strengths. First, we introduced a potentially novel clinical biomarker that is easy to collect at the bedside. Second, the study was conducted in a real-life setting in a comparably large ICU cohort. Third, we included an equally large age- and sex-matched healthy control group, which helped to establish normal reference values for the pupillary light-off reflex. Evaluating the pupillary light-off reflex in addition to the pupillary light reflex may enable a more comprehensive assessment of autonomic and higher-order brain functions after acute brain injury. However, some limitations must be acknowledged. For example, while we controlled for ambient light intensity, environmental factors such as noise and temperature were not systematically controlled for. In the same vein, patients with DoC may experience changes in intracranial pressure; they have various types and locations of brain lesions; and as stated, these patients are commonly treated with sedatives and sympathomimetics; all of which can affect pupillary function. Even though we accounted for sedation, future studies must investigate the influence of all these potentially confounding factors on the pupillary light-off reflex. Further research is also required to determine whether our cut-off for a preserved pupillary light-off reflex (i.e., within two standard deviations of that of healthy volunteers) is the optimal threshold in terms of diagnostic accuracy.

## Conclusions

We conducted a systematic investigation of the pupillary light-off reflex in the ICU. We found that this reflex can reliably distinguish between clinically unresponsive and low-responsive patients with DoC with acute brain injury. Compared to the pupillary light reflex, the absence of a normal pupillary light-off reflex appears more specific to the absence of consciousness. The pupillary light-off reflex therefore provides insights into sympathetic pupillary function (or lack thereof) and reveals indices of autonomic function after brain injury that are unavailable from the pupillary light reflex. Caveats include the incomplete knowledge of the mechanisms behind the pupillary light-off reflex in the healthy and the diseased brain. Future research is required to investigate these issues further, including possible benefits of the reflex for neuromonitoring and prognostication after acute brain injury.

## Supplementary Information

Below is the link to the electronic supplementary material.

Supplementary file1 (DOCX 32 KB)

## References

[1] Peinkhofer C, Martens P, Grand J, et al. Influence of strategic cortical infarctions on pupillary function. Front Neurol. 2018;9:916. 30420833 10.3389/fneur.2018.00916 PMC6215832

[2] Greer DM, Yang J, Scripko PD, et al. Clinical examination for prognostication in comatose cardiac arrest patients. Resuscitation. 2013;84:1546–51. 23954666 10.1016/j.resuscitation.2013.07.028 PMC4041075

[3] Loewenfeldt I. The pupil: anatomy, physiology, and clinical applications. 2nd ed. Oxford: Butterworth-Heinemann; 1999.

[4] Hunyor AP. Reflexes and the eye. Aust N Z J Ophthalmol. 1994;22:155–9. 7818872 10.1111/j.1442-9071.1994.tb01710.x

[5] Cherng Y-G, Crevecoeur F, Wang C-A. Effects of pupillary light and darkness reflex on the generation of pro- and anti-saccades. Eur J Neurosci. 2021;53:1769–82. 33314426 10.1111/ejn.15083

[6] Somani A, Lee A, Kini A, Othman B. EyeWiki–reflexes and the eye [online]. Accessed at: https://eyewiki.aao.org/Reflexes_and_the_Eye#cite_note-:0-1

[7] Loewenfeld IE. Mechanisms of reflex dilatation of the pupil–historical review and experimental analysis. Doc Ophthalmol. 1958;12:185–448. 13609524 10.1007/BF00913471

[8] Behrends M, Larson MD, Neice AE, Bokoch MP. Suppression of pupillary unrest by general Anesthesia and Propofol sedation. J Clin Monit Comput. 2019;33:317–23. 29785552 10.1007/s10877-018-0147-y

[9] Yang LL, Niemann CU, Larson MD. Mechanism of pupillary reflex dilation in awake volunteers and in organ donors. Anesthesiology. 2003;99:1281–6. 14639139 10.1097/00000542-200312000-00008

[10] Larson MD, Tayefeh F, Sessler DI, Daniel M, Noorani M. Sympathetic nervous system does not mediate reflex pupillary dilation during Desflurane Anesthesia. Anesthesiology. 1996;85:748–54. 8873544 10.1097/00000542-199610000-00009

[11] Jakobsen EW, Nersesjan V, Albrechtsen SS, et al. Brimonidine eye drops reveal diminished sympathetic pupillary tone in comatose patients with brain injury. Acta Neurochir (Wien). 2023;165:1483–94. 37014450 10.1007/s00701-023-05569-8 PMC10227128

[12] Amiri M, Fisher PM, Raimondo F, et al. Multimodal prediction of residual consciousness in the intensive care unit: the CONNECT-ME study. Brain. 2023;146:50–64. 36097353 10.1093/brain/awac335 PMC9825454

[13] Othman MH, Olsen MH, Hansen KIT, et al. Covert consciousness in acute brain injury revealed by automated pupillometry and cognitive paradigms. Neurocrit Care. 2024;41(1):218–27. 10.1007/s12028-024-01983-7. 38605221 10.1007/s12028-024-01983-7 PMC11335945

[14] Amiri M, Raimondo F, Fisher PM, et al. Multimodal prediction of 3- and 12-month outcomes in ICU patients with acute disorders of consciousness. Neurocrit Care. 2024;40:718–33. 37697124 10.1007/s12028-023-01816-z PMC10959792

[15] Wijdicks EFM, Bamlet WR, Maramattom BV, Manno EM, McClelland RL. Validation of a new coma scale: The FOUR score. Ann Neurol. 2005;58:585–93. 16178024 10.1002/ana.20611

[16] Aubinet C, Cassol H, Bodart O, et al. Simplified evaluation of consciousness disorders (SECONDs) in individuals with severe brain injury: a validation study. Ann Phys Rehabil Med. 2021;64:101432. 32992025 10.1016/j.rehab.2020.09.001

[17] Sanz LRD, Aubinet C, Cassol H, et al. SECONDs administration guidelines: a fast tool to assess consciousness in brain-injured patients. J Vis Exp. 2021;2021:1–18. 10.3791/61968 33616111

[18] Kondziella D, Bender A, Diserens K, et al. European academy of neurology guideline on the diagnosis of coma and other disorders of consciousness. Eur J Neurol. 2020;27:741–56. 32090418 10.1111/ene.14151

[19] Larson MD, Behrends M. Portable infrared pupillometry: a review. Anesth Analg. 2015;120:1242–53. 25988634 10.1213/ANE.0000000000000314

[20] Grønlund EW, Lindberg U, Fisher PM, et al. Arterial spin labeling magnetic resonance imaging for acute disorders of consciousness in the intensive care unit. Neurocrit Care. 2024. 10.1007/s12028-024-02031-0. 38918338 10.1007/s12028-024-02031-0 PMC11599417

[21] Claassen J, Doyle K, Matory A, et al. Detection of brain activation in unresponsive patients with acute brain injury. N Engl J Med. 2019;380:2497–505. 31242361 10.1056/NEJMoa1812757

[22] Egbebike J, Shen Q, Doyle K, et al. Cognitive-motor dissociation and time to functional recovery in patients with acute brain injury in the USA: a prospective observational cohort study. Lancet Neurol. 2022;21:704–13. 35841909 10.1016/S1474-4422(22)00212-5 PMC9476646

[23] Edlow BL, Chatelle C, Spencer CA, et al. Early detection of consciousness in patients with acute severe traumatic brain injury. Brain. 2017;140:2399–414. 29050383 10.1093/brain/awx176 PMC6059097

[24] Bodien YG, Allanson J, Cardone P, et al. Cognitive motor dissociation in disorders of consciousness. N Engl J Med. 2024;391:598–608. 39141852 10.1056/NEJMoa2400645 PMC7617195

[25] Kondziella D, Menon D, Helbok R, et al. A precision medicine framework for classifying patients with disorders of consciousness: advanced classification of consciousness endotypes (ACCESS). Neurocrit Care. 2021;35:27–36. 34236621 10.1007/s12028-021-01246-9

[26] Omary R, Bockisch CJ, Landau K, Kardon RH, Weber KP. Buzzing sympathetic nerves: a new test to enhance anisocoria in horner’s syndrome. Front Neurol. 2019;10:107. 30846965 10.3389/fneur.2019.00107 PMC6393781

[27] Sillito AM, Zbrozyna AW. The activity characteristics of the preganglionic pupilloconstrictor neurones. J Physiol. 1970;211:767–79. 5501060 10.1113/jphysiol.1970.sp009303 PMC1396085

[28] Samuels E, Szabadi E. Functional neuroanatomy of the noradrenergic locus coeruleus: its roles in the regulation of arousal and autonomic function part I: principles of functional organisation. Curr Neuropharmacol. 2008;6:235–53. 19506723 10.2174/157015908785777229 PMC2687936

[29] Larson MD, Kurz A, Sessler DI, Dechert M, Bjorksten AR, Tayefeh F. Alfentanil blocks reflex pupillary dilation in response to noxious stimulation but does not diminish the light reflex. Anesthesiology. 1997;87:849–55. 9357887 10.1097/00000542-199710000-00019

[30] McKay RE, Larson MD. Detection of opioid effect with pupillometry. Auton Neurosci. 2021;235:102869. 34474355 10.1016/j.autneu.2021.102869

[31] Kollars JP, Larson MD. Tolerance to miotic effects of opioids. Anesthesiology. 2005;102(3):701. 15731628 10.1097/00000542-200503000-00047

