# Supplementary material for: The Pupillary Light-Off Reflex in Acute Disorders of Consciousness
Source: Neurocrit Care. 2024 Sep 25;42(2):398–409. doi: 10.1007/s12028-024-02133-9 (PMC11950040; doi:10.1007/s12028-024-02133-9)
Supplement: Supplementary file 1 — Supplementary file1 (DOCX 32 KB) [file 12028_2024_2133_MOESM1_ESM.docx]

**Supplementary Table**

| **Supplementary Table 1: Clinical and pupillometry characteristics of unresponsive doc patients with a present light-off reflex** | | | | | | | |
| --- | --- | --- | --- | --- | --- | --- | --- |
|  | **Patient 1*** | **Patient 2*** | **Patient 3** | **Patient 4** | **Patient 5** | **Patient 6*** | **Patient 7**** |
| **Age (years)** | 45 | 24 | 42 | 51 | 68 | 81 | 57 |
| **Sex** | Male | Male | Male | Male | Male | Male | Male |
| **Admission etiology** | Cerebrovascular, hemorrhagic intracerebral | Traumatic brain injury | Traumatic Brain Injury | Cerebrovascular, hemorrhagic | Cerebrovascular, Hemorrhagic | Ischemic-anoxic brain damage | Cerebrovascular, hemorrhagic |
| **Airway management** | No invasive intervention | Tracheostomy | Orally intubated | Orally intubated | No invasive intervention | Orally intubated | Tracheostomy |
| **Sedation** | None to minimal | High to very high | High to very high | High to very high | None | High to very high | None |
| **GCS** | | | | | | | |
| Total score | 9 | 3 | 3 | 6 | 6 | 3 | 3 |
| Eye opening | 4 | 1 | 1 | 1 | 1 | 1 | 1 |
| Verbal response | 1 | 1 | 1 | 1 | 1 | 1 | 1 |
| Motor response | 4 | 1 | 1 | 4 | 4 | 1 | 1 |
| **Four Score** | | | | | | | |
| Total score | 13 | 4 | 5 | 7 | 10 | 4 | 4 |
| Eye Response | 3 | 0 | 0 | 0 | 0 | 0 | 0 |
| Motor response | 2 | 0 | 0 | 2 | 2 | 0 | 0 |
| Brainstem Reflexes | 4 | 4 | 4 | 4 | 4 | 4 | 3: Left pupil fixed and dilated |
| Respiration | 4 | 0 | 1 | 1 | 4 | 0 | 1 |
| **SECONDs** | Unresponsive Wakefulness Syndrome | Coma | Coma | Coma | Unresponsive Wakefulness Syndrome | Coma | Coma |
| **PLR-3000** | | | | | | | |
| Initial diameter (mm) | L: 2.0  R: 2.9 | L: 3.4  R: 1.9 | L: 1.6  R: 1.7 | L: 2.2  R: 2.6 | L: 2.5  R: 3 | L: 3.2  R: 1.4 | L: 5.9  R: 3.0 |
| End diameter (mm) | L: 2.4  R: 4.6 | L: 4.5  R: 2.3 | L: 2.2  R: 2.6 | L: 4.1  R: 4.3 | L: 4.4  R: 5.6 | L: 3.4  R: 1.9 | L: 6.0  R: 5.2 |
| Change in pupillary dilation diameter -diameter (mm) | L: 0.4  R: 1.7 | L: 1.1  R: 0.4 | L: 0.6  R:0.9 | L: 1.9  R: 1.7 | L: 1.9  R:2.6 | L: 0.2  R: 0.5 | L: 0.1  R: 2.2 |
| Relative change in pupillary diameter - dilation (%) | L: 20  R: 59 | L: 32  R: 21 | L: 38  R: 53 | L: 86  R: 65 | L: 76  R: 87 | L: 6  R: 36 | L: 2  R: 73 |
| Dilation velocity (mm/sec) | L: 0.60  R: 1.05 | L: 0.67  R: 0.47 | L: 0.45  R: 0.65 | L: 0.91  R: 0.78 | L: 1.18  R: 1.12 | L: 0.31  R: 0.56 | L: 0.31  R: 0.99 |
| Latency (sec) | L: 0.43  R: 0.37 | L: 0.53  R: 0.33 | L: 0.47  R: 0.40 | L: 0.50  R: 0.53 | L: 0.33  R: 0.13 | L: 0.30  R: 0.43 | L: 0.23  R: 0.47 |
| **NPi-200** | | | | | | | |
| NPi | L: 2.2  R: 4.1 | L: 4.3  R: 4.4 | L: 4.9  R: 4.8 | L: 4.5  R: 4.3 | L: 4.6  R: 4.4 | L: 3.1  R: 4.8 | L: 0.5  R: 3.6 |
| Initial diameter (mm) | L: 3.5  R: 2.6 | L: 2.3  R: 2.3 | L: 2.3  R: 2.8 | L: 4.4  R: 4.1 | L: 5.1  R: 5.5 | L: 3.3  R: 3.4 | L: 5.6  R: 6.1 |
| End diameter (mm) | L: 3.3  R: 2.3 | L: 2.0  R: 2.0 | L: 1.5  R: 1.7 | L: 2.7  R: 2.8 | L: 2.8  R: 3.2 | L: 3.0  R: 2.1 | L: 5.5  R: 4.2 |
| Change in pupillary diameter – constriction (mm) | L: 0.2  R: 0.3 | L: 0.3  R: 0.3 | L: 0.8  R: 1.1 | L: 1.7  R: 1.3 | L: 2.3  R: 2.3 | L: 0.1  R: 2.7 | L: 0.1  R: 1.9 |
| Relative change in pupillary diameter – constriction (%) | L: 4  R: 11 | L: 14  R: 12 | L: 32  R: 37 | L: 39  R: 33 | L: 45  R: 42 | L: 9  R: 39 | L: 2  R: 31 |
| Constriction velocity (mm/sec) | L: 0.19  R: 0.47 | L: 1.3  R: 1.0 | L: 1.7  R: 2.2 | L: 1.9  R: 1.2 | L: 3.3  R: 3.2 | L: 0.6  R: 1.6 | L: 0.1  R: 2.7 |
| Latency (sec) | L: 0.23  R: 0.37 | L: 0.23  R: 0.23 | L: 0.23  R: 0.23 | L: 0.23  R: 0.27 | L: 0.20  R: 0.20 | L: 0.27  R: 0.27 | L: 0.77  R: 0.27 |
| *Unilateral pupil dilation only.  **One pupil fixed and widely dilated  **Abbreviations**: Four, Full Outline of UnResponsiveness; GCS, Glasgow Coma Scale; RASS, Richmond Agitation-Sedation Scale; SECONDs, Simplified Evaluation of CONsciousness Disorders | | | | | | | |
